# Glucuronidation of drugs in humanized *UDP-glucuronosyltransferase 1* mice: Similarity with glucuronidation in human liver microsomes

**DOI:** 10.1002/prp2.2

**Published:** 2013-09-03

**Authors:** Yuki Kutsuno, Kyohei Sumida, Tomoo Itoh, Robert H Tukey, Ryoichi Fujiwara

**Affiliations:** 1 School of Pharmacy, Kitasato University5-9-1 Shirokane, Minato-ku, Tokyo, 108-8641, Japan; 2 Laboratory of Environmental Toxicology, Department of Pharmacology, University of California San DiegoLa Jolla, California

**Keywords:** drug metabolism, Humanized animal model, species difference, UDP-glucuronosyltransferase, UGT

## Abstract

Uridine 5′-diphosphate-glucuronosyltransferases (UGTs) are phase II drug-metabolizing enzymes that catalyze glucuronidation of various endogenous and exogenous substrates. Among 19 functional human UGTs, UGT1A family enzymes largely contribute to the metabolism of clinically used drugs. While the *UGT1A* locus is conserved in mammals such as humans, mice, and rats, species differences in drug glucuronidation have been reported. Recently, humanized *UGT1* mice in which the original *Ugt1* locus was disrupted and replaced with the human *UGT1* locus (*hUGT1* mice) have been developed. To evaluate the usefulness of *hUGT1* mice to predict human glucuronidation of drugs, UGT activities, and inhibitory effects on UGTs were examined in liver microsomes of *hUGT1* mice as well as in those of wild-type mice and humans. Furosemide acyl-glucuronidation was sigmoidal and best fitted to the Hill equation in *hUGT1* mice and human liver microsomes, while it was fitted to the substrate inhibition equation in mouse liver microsomes. Kinetic parameters of furosemide glucuronidation were very similar between *hUGT1* mice and human liver microsomes. The kinetics of *S*-naproxen acyl-glucuronidation and inhibitory effects of compounds on furosemide glucuronidation in *hUGT1* liver microsomes were also slightly, but similar to those in human liver microsomes, rather than in wild-type mice. While wild-type mice lack imipramine and trifluoperazine *N*-glucuronidation potential, *hUGT1* mice showed comparable *N*-glucuronidation activity to that of humans. Our data indicate that *hUGT1* mice are promising tools to predict not only in vivo human drug glucuronidation but also potential drug-drug interactions.

## Introduction

Uridine 5′-diphosphate (UDP)-glucuronosyltransferases (UGTs) are phase II drug-metabolizing enzymes that catalyze glucuronidation of compounds by transferring glucuronic acid from a co-substrate, UDP-glucuronic acid, to substrates (Dutton 1980). The family of UGTs has been classified into two subfamilies, UGT1 and UGT2, on the basis of evolutionary divergence (Mackenzie et al. 2005). The *UGT1* locus is located on chromosome 2q37 and encodes multiple unique exons 1 and common exons 2–5, producing nine functional UGT1A isoforms, UGT1A1, UGT1A3, UGT1A4, UGT1A5, UGT1A6, UGT1A7, UGT1A8, UGT1A9, and UGT1A10 (Ritter et al. 1992). In the liver, which is known as the most important tissue for detoxification, UGT1A1, UGT1A3, UGT1A4, UGT1A6, and UGT1A9 are expressed (Tukey and Strassburg 2000). The *UGT2A* and *UGT2B* genes are located on chromosome 4q13, encoding three and seven functional proteins, respectively (Mackenzie et al. 2005). The *UGT2A1* and *UGT2A2* genes are formed by exon sharing of variable first exons and common exons 2–6, similar to the mechanisms associated with the *UGT1* locus (Mackenzie et al. 2005). Meanwhile, UGT2A3 and each UGT2B are encoded by individual genes (Mackenzie et al. 2005). Each of the UGTs is expressed in a tissue-specific manner and exhibits substrate specificity (Tukey and Strassburg 2000). The UGT1A family of proteins is responsible for more than 50% of the glucuronidation potential of most prescribed drugs (Williams et al. 2004).

The *UGT1* locus is conserved in mammals such as humans, mice, and rats (Mackenzie et al. 2005). Therefore, to predict glucuronidation potential of drugs in humans, not only in vitro systems such as recombinant human UGTs have been used (Katoh et al. 2007) but also experimental animal models have been employed (Deguchi et al. 2011). Although most drugs that are glucuronidated in rodents are also conjugated in humans, species differences in the pattern of glucuronidation are extensive. One of the key differences is attributed to the fact that rodents lack a gene corresponding to human *UGT1A4*, which encodes a UGT1A4 protein that is responsible for *N*-glucuronidation of primary, secondary, and tertiary amine-containing xenobiotics such as imipramine and trifluoperazine (Nakajima et al. 2002; Uchaipichat et al. 2006). Glucuronidation of certain drugs containing carboxyl- or hydroxyl-moieties, such as furosemide and naproxen are also different among species (Rachmel and Hazelton 1986; el Mouelhi et al. 1993; Kerdpin et al. 2008). The metabolite of carboxylic acid drugs, acyl-glucuronide, is a reactive metabolite that can bind cellular proteins and DNA to form protein- and DNA-adducts, which have been associated with the development of adverse reactions such as hepatotoxicity (Koga et al. 2011). To predict and avoid human-specific drug-induced toxicities, species differences in glucuronidation need to be carefully evaluated. To overcome the species difference in drug glucuronidation, chimeric mice with humanized livers were established by transplanting human hepatocytes into an urokinase-type plasminogen activator^+/+^/severe combined immunodeficient transgenic mouse line (Katoh et al. 2008). In these mice, the replacement of their livers with human hepatocytes ranged from 80% to 90%. However, accumulating evidence has indicated that contribution of extrahepatic UGTs to metabolism of drugs could not be eliminated, as certain isoforms such as UGT1A8 and UGT1A10 are mainly expressed in the gastrointestinal tract and play an important role in drug metabolism (Mizuma 2009).

Humanized *UGT1* mice in which the original *Ugt1* locus was disrupted and replaced with the human *UGT1* locus (*hUGT1* mice) have been recently developed (Cai et al. 2010; Fujiwara et al. 2010, 2012). In this study, UGT activities along with inhibitory and heterotropic effects on UGTs were examined in liver microsomes of *hUGT1* mice, humans, and wild-type mice to evaluate the use of *hUGT1* mice to predict glucuronidation of drugs in human drug metabolism.

## Materials and Methods

### Chemicals and reagents

UDP-glucuronic acid, furosemide, estradiol, serotonin, 3′-azido-3′-deoxythymidine (AZT), and alamethicin were purchased from Sigma–Aldrich (St Louis, MO). *S*-Naproxen was purchased from Cayman Chemicals (Ann Arbour, MI). Furosemide acyl-glucuronide and *S*-Naproxen acyl-glucuronide were purchased from Toronto Research Chemicals (Toronto, ON, Canada). Imipramine and trifluoperazine were purchased from Wako Pure Chemical (Osaka, Japan). All other chemicals and solvents were of analytical grade or the highest grade commercially available. Human and male mouse liver microsomes were obtained from BD Gentest (Woburn, MA).

### Animals and preparation of liver microsomes

*Tg(UGT1*^A1*28^*)Ugt1*^*−/−*^ (*hUGT1*) mice were developed previously in a C57BL/6 background (Fujiwara et al. 2010). All animals received food and water ad libitum, and mouse handling and experimental procedures were conducted in accordance with our animal care protocol approved by Kitasato University. For tissue collections, mice were anesthetized by diethyl ether inhalation, and the liver was perfused with ice-cold 1.15% KCl. The skin and liver were rinsed in cold 1.15% KCl and stored at −80°C. Liver microsomes from male *hUGT1* mice were prepared using the following procedure. Perfused liver with 1.15% KCl was homogenized in three volumes of Tris-buffered saline (25 mmol/L Tris-HCl buffer [pH 7.4], 138 mmol/L NaCl, and 2.7 mmol/L KCl). The homogenate was centrifuged at 10,000*g* for 30 min at 4°C, and the supernatant was collected. The supernatant was centrifuged at 105,000*g* for 60 min at 4°C, and the pellet was suspended in the same buffer and used as the microsomal fraction. Protein concentrations of microsomal fractions were measured by the Bradford method using BSA as a standard (Bradford 1976).

### Enzyme assays

Furosemide and *S*-naproxen acyl-glucuronide formation was determined according to the method of Fujiwara et al. (2007b) with slight modifications. Briefly, a typical incubation mixture (200 μL of total volume) contained 100 mmol/L phosphate buffer (pH 7.4), 4 mmol/L MgCl_2_, 5 mmol/L UDP-glucuronic acid (UDPGA), 50 μg/mL alamethicin, 0.1 mg/mL liver microsomes and 50 μmol/L–5 mmol/L furosemide or 25 μmol/L–2 mmol/L *S*-naproxen. For an inhibition study, 5–200 μmol/L of estradiol, 0.05–4 mmol/L imipramine, 0.2–5 mmol/L serotonin, 10–1000 μmol/L propofol, and 500 μmol/L AZT were included in the reaction mixtures. To investigate the effects of bovine serum albumin (BSA) on the furosemide glucuronidation in the liver microsomes, 0–0.5% BSA was included in the reaction mixtures. The reaction was initiated by the addition of UDPGA after a 3-min preincubation at 37°C. After incubation at 37°C for 30 min, the reaction was terminated by addition of 200 μL of cold acetonitrile. After removal of the protein by centrifugation at 12,000*g* for 5 min, a 50-μL portion of the sample was subjected to HPLC. The enzyme assays were conducted under conditions which were linear with respect to time (<60 min) and protein content (0.4 mg/mL). As shown in Figure S1, incubation of furosemide acyl-glucuronide with liver microsomes did not decrease the amount of the acyl-glucuronide, indicating that acyl-glucuronide was stable in our enzyme assays. We used CD-1 mice liver microsomes for the control experiments; however, this is because it was demonstrated previously that liver microsomes from different mouse strains exhibited very similar kinetic parameters for drug glucuronidations (Shiratani et al. 2008).

### HPLC conditions

The formation of glucuronides was determined by the HPLC system with a LC-10AD pump (Shimadzu, Kyoto, Japan), a FP-2020 fluorescence detector (JASCO, Tokyo Japan), a SPD-10A UV detector (Shimadzu) a SIL-10A autosampler (Shimadzu), a SLC-10A system controller (Shimadzu) and a Mightysil RP-18 GP column (4.6 × 150 mm, 5 μm; Kanto Chemical, Tokyo, Japan). The mobile phases were 30% acetonitrile containing 15 mmol/L phosphate for the furosemide glucuronide, 35% acetonitrile containing 0.12% acetic acid for the *S*-naproxen glucuronide, 33% acetonitrile containing 15 mmol/L monosodium phosphate for trifluoperazine *N*-glucuronide, and 22.5% acetonitrile containing 50 mmol/L monosodium phosphate for imipramine *N*-glucuronide; the flow rate was 1.0 mL/min. Detection was accomplished using a fluorescence detector at 345-nm excitation and 450-nm emission for the furosemide glucuronide and at 230-nm excitation and 355-nm emission for the *S*-naproxen glucuronide. Detection of the imipramine and trifluoperazine *N*-glucuronide was accomplished with a UV detector at 256 nm and 205 nm, respectively. Quantification of furosemide and that of naproxen glucuronides was carried out by comparing the HPLC peak area to that of the authentic standard. The imipramine and trifluoperazine *N*-glucuronide formation was determined as reported previously (Fujiwara et al. 2007a). The retention times of furosemide glucuronide, naproxen glucuronide, imipramine glucuronide, and trifluoperazine glucuronide were 5.2, 6.5, 25.2, and 18.7 min, respectively.

### Equations

When kinetics of drug metabolism was typical and followed Michaelis–Menten kinetics, the relationship between substrate concentration and velocity was obtained by the Michaelis–Menten equation (1):

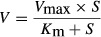
(1)where *V* is the velocity of the metabolic reaction and S is the substrate concentration. The *V*_max_ is the maximum rate of metabolism and *K*_m_ is the Michaelis constant, which is defined as the substrate concentration at 1/2 the maximum velocity. While the clearance rate is substrate concentration-dependent, the rate is constant when the substrate concentration is much smaller than *K*_m_, providing the parameter, intrinsic clearance (*CL*_int_) (eq. 2):

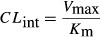
(2)

For sigmoidal kinetics, kinetic parameters were obtained by the Hill equation (3):

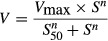
(3)where *S*_50_ is the substrate concentration showing the 1/2 *V*_max_ and *n* is the Hill coefficient. While the clearance rate is substrate concentration-dependent, the maximum clearance rate, *CL*_max_, can be described by equation (4): (Houston and Kenworthy 2000)

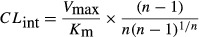
(4)

Substrate inhibition kinetics was analyzed by equation (5):

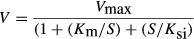
(5)where *K*_si_ is the constant describing the substrate inhibition interaction.

IC_50_ values were directly determined from linear regression.

## Results

### Furosemide glucuronidation in liver microsomes from *hUGT1* mice, human, and regular mice

**Figure 1:**
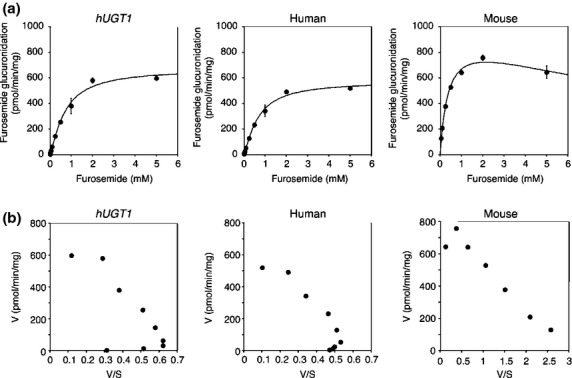
Kinetic analyses of furosemide acyl-glucuronide formation in liver microsomes. The substrate concentration-velocity curves (A) and Eadie–Hofstee plots (B) of the furosemide glucuronide formation are shown. Pooled liver microsomes of *hUGT1* mice, humans, and regular mice were incubated with 50 μmol/L to 5 mmol/L furosemide and 5 mmol/L UDP-glucuronic acid at 37°C for 30 min. In the substrate concentration-velocity curves, data are the means ± SD of three independent determinations. In the Eadie–Hofstee plots, each data point represents the mean of three independent experiments.

Furosemide is a drug that is subject to species-different glucuronidation (Rachmel and Hazelton 1986; Kerdpin et al. 2008). To examine whether furosemide glucuronidation in *hUGT1* mice is similar to that in humans, liver microsomes were prepared from adult *hUGT1* mice and furosemide glucuronidation was determined. The furosemide acyl-glucuronide formation by the liver microsomes from *hUGT1* mice followed the Hill equation (Fig. 1A), yielding *S*_50_ = 715 μmol/L, *V*_max_ = 673 pmol/min/mg, Hill coefficient, *n* = 1.30, and *CL*_max_ = 0.55 μL/min/mg (Table 1). Eadie–Hofstee plots were not linear (Fig. 1B), supporting the fact that the furosemide glucuronidation in liver microsomes from *hUGT1* mice was sigmoidal. In human liver microsomes, the furosemide acyl-glucuronide formation also followed the Hill equation (Fig. 1A) as Eadie–Hofstee plots were not linear (Fig. 1B), yielding *S*_50_ = 681 μmol/L, and *V*_max_ = 576 pmol/min/mg, Hill coefficient = 1.30, *CL*_max_ = 0.50 μL/min/mg (Table 1). In contrast, the furosemide acyl-glucuronide formation by mouse liver microsomes followed the Michaelis–Menten equation with a substrate inhibition as Eadie–Hofstee plots curved at a higher substrate concentration (Fig. 1A and B), yielding *K*_m_ = 405 μmol/L, *V*_max_ = 998 pmol/min/mg, *K*_i_ = 12.2 mmol/L, and *CL*_int_ = 2.47 μL/min/mg (Table 1). These data confirmed that furosemide was species-differently glucuronidated in liver microsomes, as the kinetics was sigmoidal in humans, while it was typical in mice. Furosemide glucuronidation in *hUGT1* mice showed sigmoidal kinetics, similar to the kinetic pattern formed in human liver microsomes. The clearance values predicted using *hUGT1* liver and human liver microsomes were similar, 0.55 and 0.50 μL/min/mg, respectively. In contrast, the clearance value of wild-type mice was dramatically higher, 2.47 μL/min/mg. These results indicate that *hUGT1* mice can be used to predict furosemide glucuronidation in humans, quantitatively.

**Table 1:** Kinetic parameters of furosemide glucuronidation in liver microsomes

Liver microsomes	Equation	*K*_m_ or *S*_50_ (μmol/L)	*n*	*V*_max_	*K*_i_	*CL*_int_ or *CL*_max_ (μL/min/mg)
Humanized *UGT1* mice	Hill	715 ± 103	1.30 ± 0.14	673 ± 21 pmol/min/mg	—	0.55 ± 0.06
Human	Hill	681 ± 124	1.30 ± 0.12	576 ± 21 pmol/min/mg	—	0.50 ± 0.07
Regular mice	Substrate inhibition	405 ± 14	—	998 ± 40 pmol/min/mg	12.2 ± 3.8 mmol/L	2.47 ± 0.2

### *S*-Naproxen glucuronidation in liver microsomes from *hUGT1* mice, human, and mice

**Figure 2:**
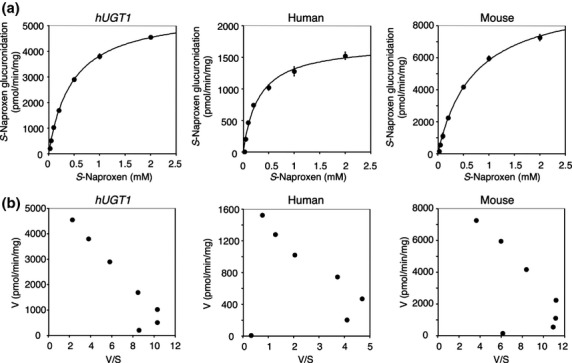
Kinetic analyses of *S*-naproxen acyl-glucuronide formation in liver microsomes. The substrate concentration-velocity curves (A) and Eadie–Hofstee plots (B) of the *S*-naproxen glucuronide formation are shown. Pooled liver microsomes of *hUGT1* mice, humans, and regular mice were incubated with 25 μmol/L to 2 mmol/L *S*-naproxen and 5 mmol/L UDP-glucuronic acid at 37°C for 30 min. In the substrate concentration-velocity curves, data are the means ± SD of three independent determinations. In the Eadie–Hofstee plots, each data point represents the mean of three independent experiments.

It has been reported that naproxen was glucuronidated differently when comparing metabolism between human and rodents (el Mouelhi et al. 1993). *S*-Naproxen glucuronidation in *hUGT1* mice, human and wild-type mice liver microsomes was determined and the kinetics was analyzed. Although the *S*-naproxen acyl-glucuronide formation by the liver microsomes from *hUGT1* mice was best fitted to the Hill equation as the Eadie–Hofstee plots were not linear at lower substrate concentrations, the Hill coefficient was 1.0, indicating that there is no cooperativity (Fig. 2). Therefore, the kinetic parameters of *S*-naproxen acyl-glucuronide formation were obtained by the Michaelis–Menten equation. In the liver microsomes from *hUGT1* mice, the *S*-naproxen acyl-glucuronide formation yielded *K*_m_ = 465 μmol/L, *V*_max_ = 5.60 nmol/min/mg, and *CL*_int_ = 12.0 μL/min/mg (Table 2). In human liver microsomes, the *S*-naproxen acyl-glucuronide formation also followed the Michaelis–Menten equation (Fig. 2), yielding *K*_m_ = 308 μmol/L, *V*_max_ = 1.72 nmol/min/mg, and *CL*_int_ = 5.6 μL/min/mg (Table 2). The *S*-naproxen acyl-glucuronide formation by wild-type mouse liver microsomes followed the Michaelis–Menten equation, yielding *K*_m_ = 703 μmol/L, *V*_max_ = 10.0 nmol/min/mg, and *CL*_int_ = 14.2 μL/min/mg (Table 2). The kinetic parameters obtained in liver microsomes from *hUGT1* mice were slightly closer to the parameters in human liver microsomes than to those in mouse liver microsomes.

**Table 2:** Kinetic parameters of *S*-naproxen glucuronidation in liver microsomes

Liver microsomes	Equation	*K*_m_ (μmol/L)	*V*_max_ (nmol/min/mg)	Clearance (*CL*_int_) (μL/min/mg)
Humanized *UGT1* mice	Michaelis–Menten	465 ± 14	5.60 ± 0.09	12.0 ± 0.2
Human	Michaelis–Menten	308 ± 30	1.72 ± 0.11	5.6 ± 0.2
Regular mice	Michaelis–Menten	703 ± 54	10.0 ± 0.4	14.2 ± 0.5

### Inhibitory and heterotropic effects on furosemide glucuronidation in liver microsomes from *hUGT1* mice, human, and mice

**Figure 3:**
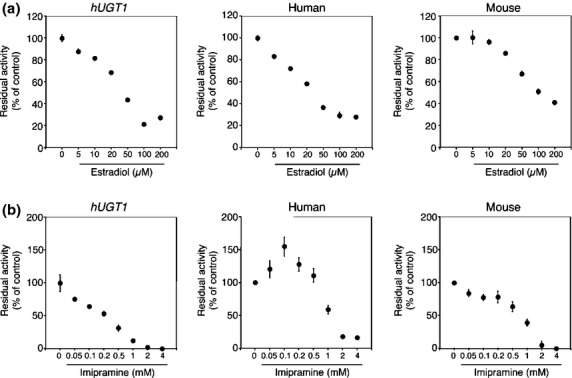
Inhibitory effects of estradiol (A) and imipramine (B) on furosemide acyl-glucuronide formation by liver microsomes of *hUGT1* mice, humans, and regular mice. Residual activities were shown compared to the activity obtained in the absence of inhibitors. Data are the means ± SD of three independent determinations.

**Figure 4:**
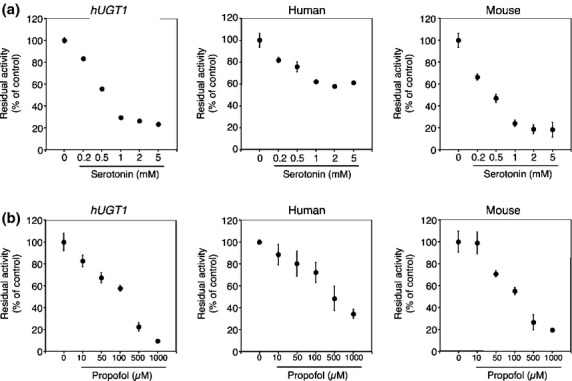
Inhibitory effects of serotonin (A) and propofol (B) on furosemide acyl-glucuronide formation by liver microsomes of *hUGT1* mice, humans, and regular mice. Residual activities were shown compared to the activity obtained in the absence of inhibitors. Data are the means ± SD of three independent determinations.

**Figure 5:**
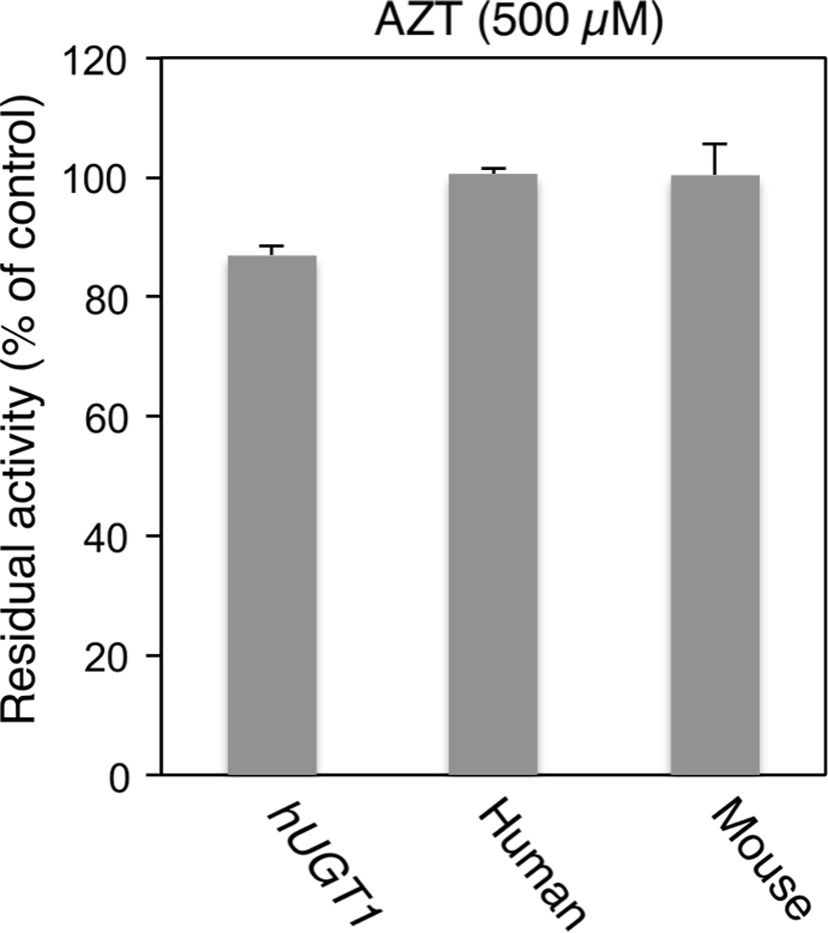
Inhibitory effects of AZT on furosemide acyl-glucuronide formation by liver microsomes of *hUGT1* mice, humans, and regular mice. Residual activities were shown compared to the activity obtained in the absence of AZT. Data are the means ± SD of three independent determinations.

To investigate the species difference in inhibitory effects on furosemide glucuronidation, inhibitory effects of selective inhibitors, estradiol (UGT1A1), imipramine (UGT1A4), serotonin (UGT1A6), propofol (UGT1A9), and AZT (UGT2B7) were examined in liver microsomes from *hUGT1* mice, humans, and wild-type mice. Among these inhibitors, estradiol exhibited the most potency inhibition toward furosemide glucuronidation, as the IC_50_ values were 32.5 μmol/L, 31.7 μmol/L, and 110.8 μmol/L in liver microsomes from *hUGT1* mice, humans, and mice, respectively (Fig. 3A and Table 3). Propofol moderately inhibited furosemide glucuronidation with IC_50_ values of 186 μmol/L, 474 μmol/L, and 169 μmol/L in liver microsomes from *hUGT1* mice, humans, and mice (Fig. 4B). The inhibitory effects of imipramine and serotonin toward furosemide glucuronidation was slight; their IC_50_ values were 0.25 mmol/L and 0.61 mmol/L in *hUGT1* mice, 1.2 mmol/L and >5 mmol/L in humans, and 0.78 mmol/L and 0.45 mmol/L in regular mice, respectively (Figs. 3B and 4A). AZT did not inhibit furosemide glucuronidation in any species (Fig. 5). These data demonstrated that in most cases, inhibitory effects toward furosemide glucuronidation in *hUGT1* mice were similar to those in humans. Especially, the IC_50_ values of estradiol were comparable between *hUGT1* mice and humans, indicating that *hUGT1* mice can be used to examine the inhibitory effects of compounds on the glucuronidation of drugs in humans.

**Table 3:** IC_50_ values for the inhibition of furosemide glucuronidation in liver microsomes

Liver microsomes	Estradiol (μmol/L)	Imipramine (mmol/L)	Serotonin (mmol/L)	Propofol (μmol/L)	AZT (μmol/L)
Humanized *UGT1* mice	32.5	0.25	0.61	186	>500
Human	31.7	1.2	>5	474	>500
Regular mice	110.8	0.78	0.45	169	>500

It should be noted that a lower concentration of imipramine (100 μmol/L) activated the furosemide glucuronidation in human liver microsomes. With 100 μmol/L imipramine, UGT activity was increased 1.5-fold compared to that in the absence of imipramine (Fig. 3B). In contrast, in the liver microsomes of *hUGT1* mice and wild-type mice, such heterotropic activation of furosemide glucuronidation by imipramine was not observed.

### Imipramine and trifluoperazine glucuronidation in liver microsomes from *hUGT1* mice, human, and mice

**Figure 6:**
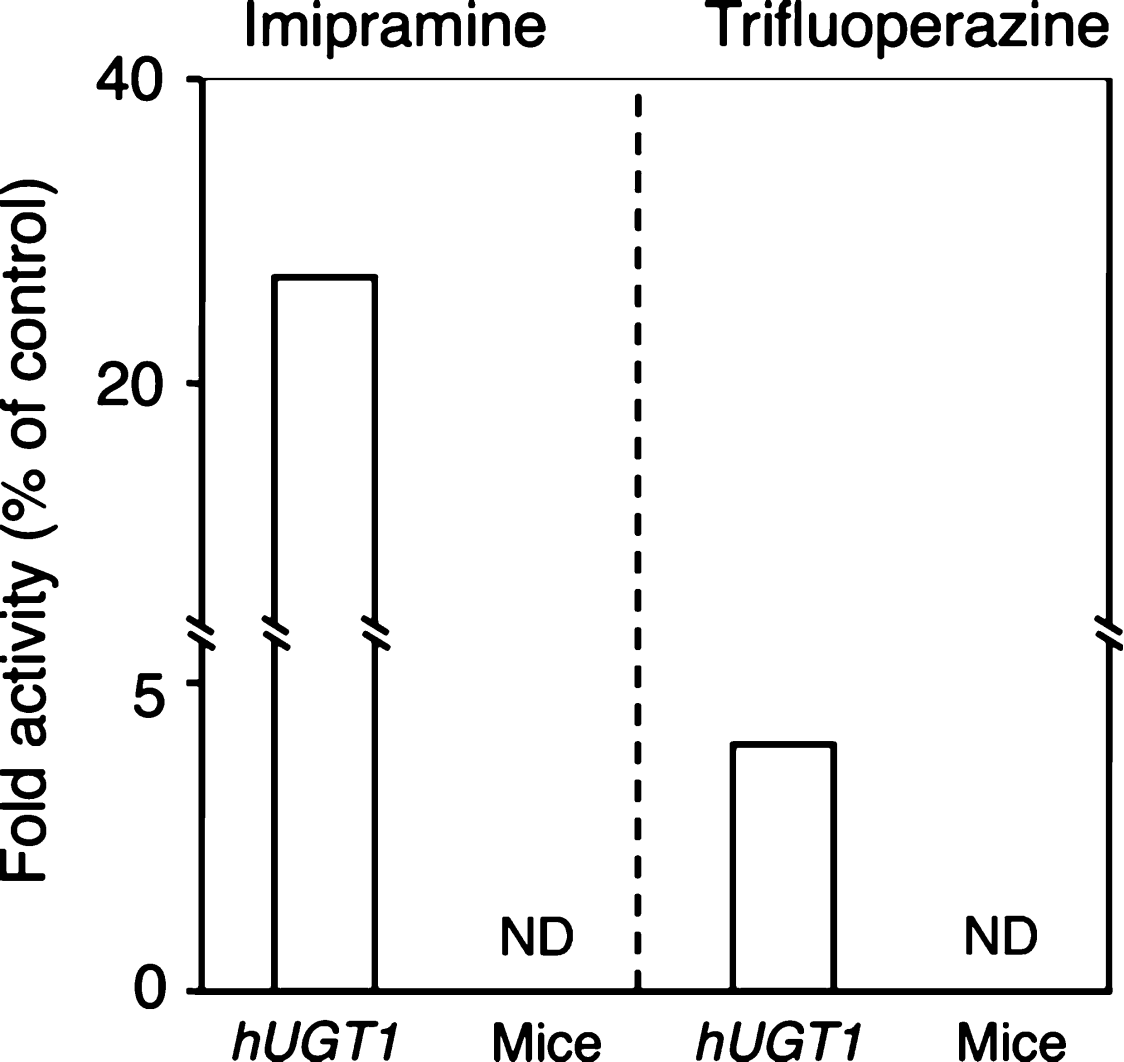
Imipramine and trifluoperazine *N*-glucuronidation in liver microsomes of *hUGT1* mice, humans, and regular mice. Fold activities were shown compared to the activity obtained in human liver microsomes. Each column represents the mean of three independent determinations. ND: not detected.

While other UGT isoforms such as UGT1A3 can catalyze the *N*-glucuronidation of primary, secondary, and tertiary amine-containing compounds (Green et al. 1998), UGT1A4 has been known as the primary enzyme responsible for *N*-glucuronidation (Kaivosaari et al. 2011). Since rodents lack the human *UGT1A4* homologue gene, regular experimental mice and rats exhibit less or no *N*-glucuronidation of activity (Al-Zoughool and Talaska 2005; Shiratani et al. 2008). To examine the potential usefulness of *hUGT1* mice in predicting *N*-glucuronidation of drugs in humans, *N*-glucuronide formation of imipramine and that of trifluoperazine, which are substrates for human UGT1A4, were determined in liver microsomes from *hUGT1* mice, humans, and wild-type mice. While imipramine and trifluoperazine *N*-glucuronide formations were observed in human liver microsomes, those metabolites were not detected in mouse liver microsomes (Fig. 6). In the liver microsomes from *hUGT1* mice, however, comparable amounts of imipramine *N*-glucuronide formation and a lesser but detectable amount of trifluoperazine *N*-glucuronide formation were observed (Fig. 6). These data indicate that *hUGT1* mice have the potential capacity to form *N*-glucuronides of drugs.

### Effects of BSA on furosemide glucuronidation in liver microsomes

It has been demonstrated that fatty acids included in liver microsomes inhibit glucuronidation reaction in vitro and that albumin can attenuate the inhibitory effects of fatty acids (Rowland et al. 2007). To investigate the species difference in the effects of albumin on the microsomal drug glucuronidation, 0–0.5% of BSA was included in the reaction mixtures and the enzyme activities were determined. As reported previously, BSA concentration-dependently activated the glucuronidation of drug in human liver microsomes (Fig. S2). Interestingly, such activation was similarly observed in liver microsomes from *hUGT1* mice and control mice (Fig. S2). This indicates that there is no species difference in the effect of BSA on furosemide glucuronidation, and that BSA similarly activates microsomal glucuronidation in *hUGT1* mice.

## Discussion

Various efforts have been undertaken to predict human glucuronidation of drugs to optimize clinical dosages of drugs and also to avoid in vivo drug-drug interactions that can significantly increase or decrease the area under the plasma drug concentration-time curve (AUC). Among 19 functional human UGTs, the UGT1A family of proteins plays a key role in the metabolism of clinically used drugs (Williams et al. 2004). UGT1A enzymes expressed in tissue culture have been used to examine substrate specificity (Zhang et al. 2005); however, recent reports have demonstrated that clearance values calculated from in vitro data using UGT-expressing cells did not correlate well to those observed in vivo (Lin and Wong 2002), possibly due to the presence of protein–protein interactions between the UGT isoforms (Fujiwara et al. 2007a,b; Operaña and Tukey 2007). While primary cultured human hepatocytes can mimic in vivo drug glucuronidation (Miners et al. 2006), these hepatocytes are not only expensive and inconvenient to culture but also inconsistent, exhibiting different metabolic properties in each study, which can potentially be attributed to genetic polymorphisms or storage conditions. Animal scale-up is an alternative method for predicting in vivo human drug metabolism. A recent report by Deguchi et al. (2011) demonstrated that animal scale-up with monkeys, rather than with mice, could be a reliable method in predicting human pharmacokinetics of UGT substrates. However, due to their convenience, rodents are still the most extensively used animals in research.

The fundamental obstacle to utilizing rodents to predict human drug glucuronidation is that they lack a human *UGT1A4* homologue gene, which encodes a primary UGT isoform responsible for *N*-glucuronidation. In addition, there are significant differences in substrate specificity attributed toward rodent and human homologs. Since *hUGT1* mice carry the entire human *UGT1* locus including *UGT1A4*, they can form *N*-glucuronides of imipramine and trifluoperazine in liver microsomes (Fig. 6), though *N*-glucuronidation activity is comparable or lower than that in human liver microsomes. The transgene introduced into *hUGT1* mice contains promoter regions of each UGT1A isoform; therefore, treatment of *hUGT1* mice with several UGT1A4 inducers would result in increased UGT1A4-catalyzed *N*-glucuronidations of drugs in the *hUGT1* mice, as already demonstrated in human *UGT1* transgenic mice (Chen et al. 2005). In addition to the *N*-glucuronidation of imipramine and trifluoperazine, the metabolic profile of the microsomal glucuronidation activity toward furosemide in *hUGT1* mice was very similar to that in human liver microsomes, considering the identical clearance parameters and Eadie–Hofstee plots in *hUGT1* mice and humans (Fig. 1 and Table 1). In contrast, similarity of *S*-naproxen glucuronidation between *hUGT1* mice and humans was not significant, although the kinetic parameters of *S*-naproxen glucuronidation in *hUGT1* mice were slightly closer to the parameters in humans than to those in mice (Table 2). This might be explained by the contribution of UGT2B family enzymes to *S*-naproxen glucuronidation. It has been shown that UGT1A and UGT2B proteins can glucuronidate *S*-naproxen (Bowalgaha et al. 2005). In humans, multiple *UGT2B* genes encode UGT2B4, UGT2B7, UGT2B10, UGT2B11, UGT2B15, UGT2B17, and UGT2B28 proteins. In mice, seven *Ugt2b* genes include Ugt2b1, 2b5, 2b34, 2b35, 2b36, 2b37, and 2b38. The species difference in the function of human UGT2B and mouse Ugt2b family enzymes has not been fully investigated. However, it has been reported that morphine 3-glucuronidation, which is specifically catalyzed by human UGT2B7 in human liver microsomes was significantly lower than the activity in mice, as the *V*_max_ values in humans and mice were 2.7 and 19 nmol/min/mg, respectively (Court et al. 2003; Shiratani et al. 2008). Therefore, humanized *UGT* mice, in which not only *Ugt1* but also *Ugt2* genes can be replaced with human *UGT1A* and *UGT2B* genes, will become valuable animal models for predicting human glucuronidation of drugs.

While the kinetics of furosemide was similar between *hUGT1* mice and humans, it should be noted that the hetero-activation of UGT1A-catalyzed furosemide glucuronidation by a lower concentration of imipramine was not observed in *hUGT1* mice, which is in contradiction to the heterotropic activation observed in human liver microsomes (Fig. 3). Various compounds, including anthraflavic acid, 17 alpha-ethynylestradiol, 2-amino-1-methyl-6-phenylimidazo(4,5-b)pyridine, androstanediol, propofol, daidzein, 4-methylumbelliferone, and estradiol, have been reported as heterotropic activators of human UGT-catalyzed glucuronidation (Pfeiffer et al. 2006; Mano et al. 2004: Yamanaka et al. 2005; Williams et al. 2002). Interestingly, those heterotropic activations were reported in human liver microsomes and human UGT expressing cells, but not in other species such as mouse liver microsomes. Our data revealed that even though human UGT1A proteins were expressed in the mouse liver, such activation did not occur, indicating that differences in amino acid sequence, as well as factors such as membrane topology and latency of UGTs might be associated with the species different appearance of heterotropic activation. Indeed, latency of UGT activity has been reported to be different in humans and mice, as detergents, Brij 58 and CHAPS, only increased UGT activities by 9–66% in human liver microsomes, whereas activity increased by 439–563% in mouse liver microsomes (Court and Greenblatt 1997).

Human liver microsomes are commercially available and are a convenient tool to study human glucuronidation in in vitro research. However, predicted clearance values of glucuronidation from in vitro data utilizing human liver microsomes do not correlate well to observed in vivo clearances of drugs. While animal scale-up is an alternative method for predicting human drug glucuronidation, species difference in UGT activities can be a barrier for accurate prediction of in vivo glucuronidation of drugs in humans. In this study, we demonstrated that drug glucuronidation in *hUGT1* mice was similar to that in humans. Furthermore, inhibitory effects of estradiol on UGT activities in *hUGT1* mice were comparable to those in humans. This indicates that in vivo and in vitro studies utilizing *hUGT1* mice are promising for predicting not only in vivo human drug glucuronidations but also potential drug–drug interactions.

## Abbreviations

UGT: UDP-glucuronosyltransferase
HPLC: high performance liquid chromatography
*hUGT1*: humanized *UGT1*
AZT: 3′-azido-3′-deoxythymidine
AUC: area under the plasma drug concentration-time curve
BSA: bovine serum albumin

## Supporting Information

**Figure S1.** Incubation of furosemide glucuronide with microsomes did not decrease the amount of the acyl-glucuronide. **Figure S2.** Effects of BSA on furosemide glucuronidation in liver microsomes.
