# Supplementary material for: Glucuronidation of drugs in humanized UDP-glucuronosyltransferase 1 mice: Similarity with glucuronidation in human liver microsomes
Source: Pharmacol Res Perspect. 2013 Sep 3;1(1):e00002. doi: 10.1002/prp2.2 (PMC4184567; doi:10.1002/prp2.2)
Supplement: Supplementary file 1 — Figure S1. Incubation of furosemide glucuronide with microsomes did not decrease the amount of the acyl-glucuronide. Figure S2. Effects of BSA on furosemide glucuronidation in liver microsomes. [file prp20001-e00002-sd1.pdf]

Supplemental data

Glucuronidation of drugs in humanized *UDP-glucuronosyltransferase 1* mice: Similarity with glucuronidation in human liver microsomes

Yuki Kutsuno, Kyohei Sumida, Tomoo Itoh, Robert H. Tukey, and Ryoichi Fujiwara

Supplemental Figure 1

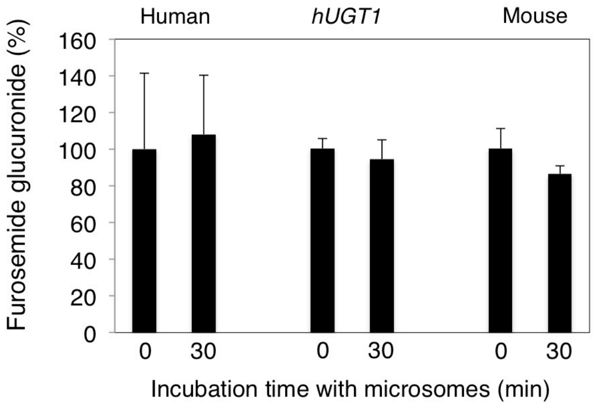

Supplemental Figure 1. Incubation of furosemide glucuronide with microsomes did not decrease the amount of the acyl-glucuronide. Furosemide acyl-glucuronide (1 μM) was incubated with liver microsomes (0.1 mg/mL) for 30 min. Data are the means ± S.D. of three independent determinations.

Supplemental Figure 2

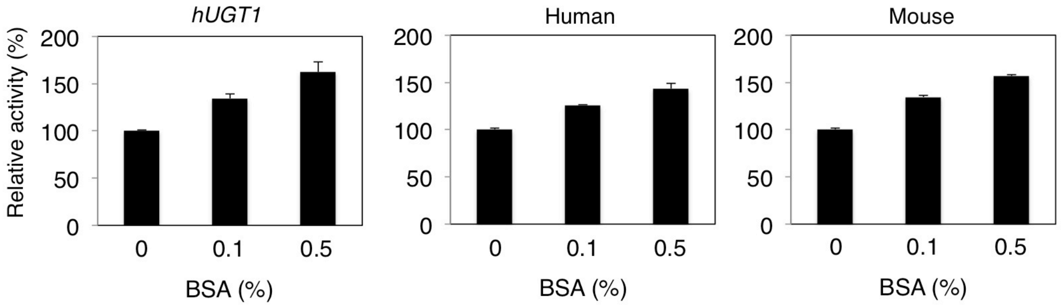

Supplemental Figure 2. Effects of BSA on furosemide glucuronidation in liver microsomes. BSA was included in the reaction mixtures (0-0.5%) and the enzyme activities were determined. Data are the means ± S.D. of three independent determinations.
